# Incomplete methylation of a germ cell tumor (Seminoma) in a Prader‐Willi male

**DOI:** 10.1002/mgg3.448

**Published:** 2018-07-12

**Authors:** Talia Eldar‐Geva, Varda Gross‐Tsur, Harry J. Hirsch, Gheona Altarescu, Reeval Segal, Sharon Zeligson, Eliahu Golomb, Silvina Epsztejn‐Litman, Rachel Eiges

**Affiliations:** ^1^ Reproductive Endocrinology and Genetics Unit Department of Obstetrics and Gynecology Shaare Zedek Medical Center Jerusalem Israel; ^2^ Neuropediatric Unit Department of Pediatrics Israel Multidisciplinary PraderWilli Syndrome Clinic Shaare Zedek Medical Center Jerusalem Israel; ^3^ The Hebrew University Faculty of Medicine Jerusalem Israel; ^4^ Zohar PGD Lab Medical Genetics Institute Shaare Zedek Medical Center Jerusalem Israel; ^5^ Department of Pathology Shaare Zedek Medical Center Hebrew University Jerusalem Israel; ^6^ Stem Cell Research Laboratory Medical Genetics Institute Shaare Zedek Medical Center Jerusalem Israel

**Keywords:** DNA methylation, genomic imprinting, induced pluripotent stem cells, Prader‐Willi syndrome, seminoma

## Abstract

**Background:**

Prader‐Willi syndrome (PWS) is a multisystem genetic disorder characterized by lack of satiety leading to morbid obesity, variable degrees of mental retardation, behavior disorders, short stature, and hypogonadism. The underlying genetic cause for PWS is an imprinting defect resulting from a lack of expression of several paternally inherited genes embedded within the 15q11.2‐q13 region. Although the clinical expression of hypogonadism in PWS is variable, there are no known cases of fertility in PWS men. In this paper, we described a pure, nearly diploid seminoma in an apparently 32 year‐old infertile man with PWS due to maternal uniparental disomy (UPD) on chromosome 15. The development of a germ cell tumor in this subject was an unanticipated result. The aim of this study was to explore the origin of the germ cell tumor in this PWS male patient.

**Methods:**

To explain the origin of the germ cell tumor (seminoma) in our PWS patient we have characterized the tumor for cell morphology and tumor type by pathological examination (H&E and immuno‐stainings), evaluated its karyotype by chromosomal microarray analysis and confirmed its UPD origin by haplotype analysis. In addition, DNA methylation status of the PWS‐ and H19‐ imprinting centers in wild‐type and affected fibroblasts, patient derived induced pluripotent stem cells (iPSCs), and PWS seminoma were determined by bisulfite DNA colony sequencing.

**Results:**

To explain the apparent contradiction between the existence of a germ cell tumor and hypogonadism we first confirmed the germ cell origin of the tumor. Next, we determined the tumor chromosomal composition, and validated the presence of a maternal UPD in all examined cell types from this patient. Finally, we characterized the maternal imprints in the PWS and H19 imprinting centers in the tumor and compared them with patient's fibroblasts and iPSCs derived from them. Unpredictably, methylation was reduced to 50% in the tumor, while preserved in the other cell types.

**Conclusion:**

We infer from this assay that the loss of methylation in the PWS‐IC specifically in the tumor of our patient is most likely a locus‐specific event resulting from imprint relaxation rather than from general resetting of the imprints throughout the genome during germ line specification.

## 1 INTRODUCTION

Prader‐Willi syndrome (PWS) is a multisystem genetic disorder with an incidence ranging from 1:10,000 to 1:20,000 births (OMIM #176270). The major clinical features include lack of satiety leading to morbid obesity, variable degrees of mental retardation, behavior disorders, short stature, and hypogonadism (Cassidy & Driscoll, 2009). The underlying genetic cause for PWS is an imprinting defect resulting from a lack of expression of several paternally inherited genes embedded within the 15q11.2‐q13 region. There are three genetic subtypes: deletions of paternally inherited genes (65%‐75% of cases), maternal uniparental disomy (UPD) (20%‐30%), and an imprinting defect in the PWS imprinting center (1%–5%) of PWS patients.

Although the clinical expression of hypogonadism in PWS is variable, there are no known cases of fertility in PWS men (Crinò et al., 2003) (Hirsch, Eldar‐Geva, Benarroch, Rubinstein, & Gross‐Tsur, 2009). Most have primary testicular dysfunction with markedly elevated FSH levels, while hypogonadotrophic hypogonadism is less common (Hirsch et al., 2009).

Testicular germ cell tumors are thought to originate from primordial germ cells which undergo maturation arrest and not from poorly differentiated somatic cells (Netto et al., 2008) (Looijenga et al., 2000) (Sell & Pierce, 1994). Seminoma, the most common pure germ cell tumor of the testis, appears to develop from gonadal stem cells which have reached a more mature stage of differentiation, and present a more homogeneous phenotype compared to other germ cell tumors.

Except for myeloid leukemia, malignancies appear to be relatively rare in PWS perhaps because of a high prevalence of growth hormone deficiency and low insulin‐like growth factor 1 (IGF1) (Davies et al., 2003) (Shevah & Laron, 2007). Seminoma was reported in only one other PWS man, aged 40 years, who presented with testicular swelling along with an abdominal mass (Robinson & Jones, 1990). Premalignant intratubular germ cell neoplasia (ITGCN) was found in a cryptorchidic abdominal testis of a 9 year‐old boy with PWS due to paternal deletion (Jaffray, Moore, & Dickson, 1999).

We now report a 32 year‐old man with PWS due to UPD who was found to have a testicular mass on routine examination. The tumor pathology showed a diagnosis of seminoma of germ cell origin, despite hypogonadotrophic hypogonadism in our patient and known infertility in PWS men. Unexpectedly, methylation levels were reduced to 50%, in contrast with complete methylation of his skin fibroblasts and induced pluripotent stem cells (iPSCs) derived from them.

## 2 MATERIALS AND METHODS

This study was approved by the institutional review board of Shaare Zedek Medical Center (IRB 88/11 provided by the Institution's ethic committee) and a signed informed consent was obtained from the patient and his parents.

A 32 year‐old man with PWS due to UPD was found to have enlarged left testicle on routine examination. Past history included severe hypotonia, poor sucking, and bilateral cryptorchidism that were noted in infancy. At age 2 years, he underwent bilateral orchidopexy. He did not receive growth hormone, human chorionic gonadotropins (hCG), testosterone, or other hormonal treatment. When first seen in our multidisciplinary PWS clinic at age 27.9 years, his height was 164.0 cm, weight 88.6 kg, and BMI 32.4 kg/m^2^. His left testis was palpable in the scrotum while the right testis was palpable in the inguinal canal. Each testis was approximately 2.5 cm (maximum length) and 1 cm diameter. Penile stretched length was 8 cm and pubic hair was Tanner stage 4. Hormone levels included LH 0.2mIU/ml (N: 1.5‐9), FSH 1.2mIU/ml (N: 2–9.2), testosterone 1.04 ng/ml (N: 2.7–10.7 ng/ml), prolactin 38.2 pg/ml (N: 3‐25), AMH 32.3 ng/ml (N: 3–20), and inhibin B <10 pg/ml (N: 90‐300).

On a routine examination at the age of 32 years, his right testis was firm and measured 3 × 1.6X1 cm, while the left one measured 4.2 × 2.5 × 2.7 cm. An ultrasound examination showed right atrophic testis with no localized lesion. The left testis was very vascular with small calcifications and hyper‐echogenic lesions suggestive of a testicular tumor. Abdominal CT scan showed no metastatic lesions. Serum: hCG was undetectable and alpha‐fetoprotein was normal (1.4 ng/ml). A left orchiectomy was performed. On pathological examination a pure seminoma was diagnosed which occupied almost the entire testis. The area around the tumor showed intratubular germ cell neoplasia. No normal tissue was found, and therefore spermatogenesis assessment was not possible.

### 2.1 Tumor haplotype analysis

On pathological examination a pure seminoma, confined to the testis, was diagnosed. The tumor occupied almost the entire testis, with marginal intratubular germ cell neoplasia around it. No normal tissue was found, and therefore spermatogenesis assessment was not possible.

### 2.2 DNA marker analysis

Two informative polymorphic markers on chromosome 15 (D15S122, D15S127) have been identified between the patient and his parents. Haplotype analysis was carried out on genomic DNA extracted from the seminoma, the patient's iPSCs and DNA extracted from blood samples of the parents.

### 2.3 Tumor chromosomal profile

Chromosomal microarray analysis (CMA) was performed using an Affymetrix CytoScan 750K array after DNA was PCR amplified, fragmented, and labeled according to the Affymetrix manufacture protocol.

### 2.4 Derivation of iPSCs from skin fibroblasts and seminoma

Four transcription reprogramming retro‐viral vectors expressing *OCT4, SOX2, KLF4,* and *c‐MYC*, were individually packaged in 293T cells. Infectious viruses were collected 24 and 48 hours post‐transfection and immediately added to primary fibroblasts derived from the patient. Four days following infection the cells were placed on mytomycin C‐treated MEFs and maintained in hESC media. Manual isolation of single clones was carried out approximately 30 days post‐transfection, resulting in stable cell lines with hESC‐like morphology.

### 2.5 Methylation analysis by bisulfite DNA sequencing

Methylation status of the PW‐IC and H19‐IC regions in wild‐type and affected fibroblasts, iPSCs, and PWS seminoma were determined by bisulfite DNA colony sequencing. Genomic DNA (2 μg) was modified by bisulfite treatment (EZ DNA methylation Kit™, Zymo Research) and amplified by nested PCR using FastStart™ DNA polymerase (Roche). Amplified products were cloned and single colonies were analyzed for CpG methylation by direct sequencing (ABI 3130). Primers sequences were as follows: PW‐IC outer primers: TCCAAAACAAAAACTTTAAAACCCAAATTC and AGGTTTTTTTTTATTGTAATAGTGTTGTGGGG and nested primers: TCAATACTCCAAATCCTAAAAACTTAAAATATC and TGTGGGGTTTTAGGGGTTTAGTAGTTTTTTTTTTTTA (341 bp final products); H19‐IC outer primers: TTTTTGGTAGGTATAGAGTT and AAACCATAACACTAAAACCC and nested primers: TGTATAGTATATGGGTATTTTTGGAGGTTT and TCCCATAAATATCCTATTCCCAAATAACC (231 bp final products).

## 3 RESULTS

**Figure 1:**
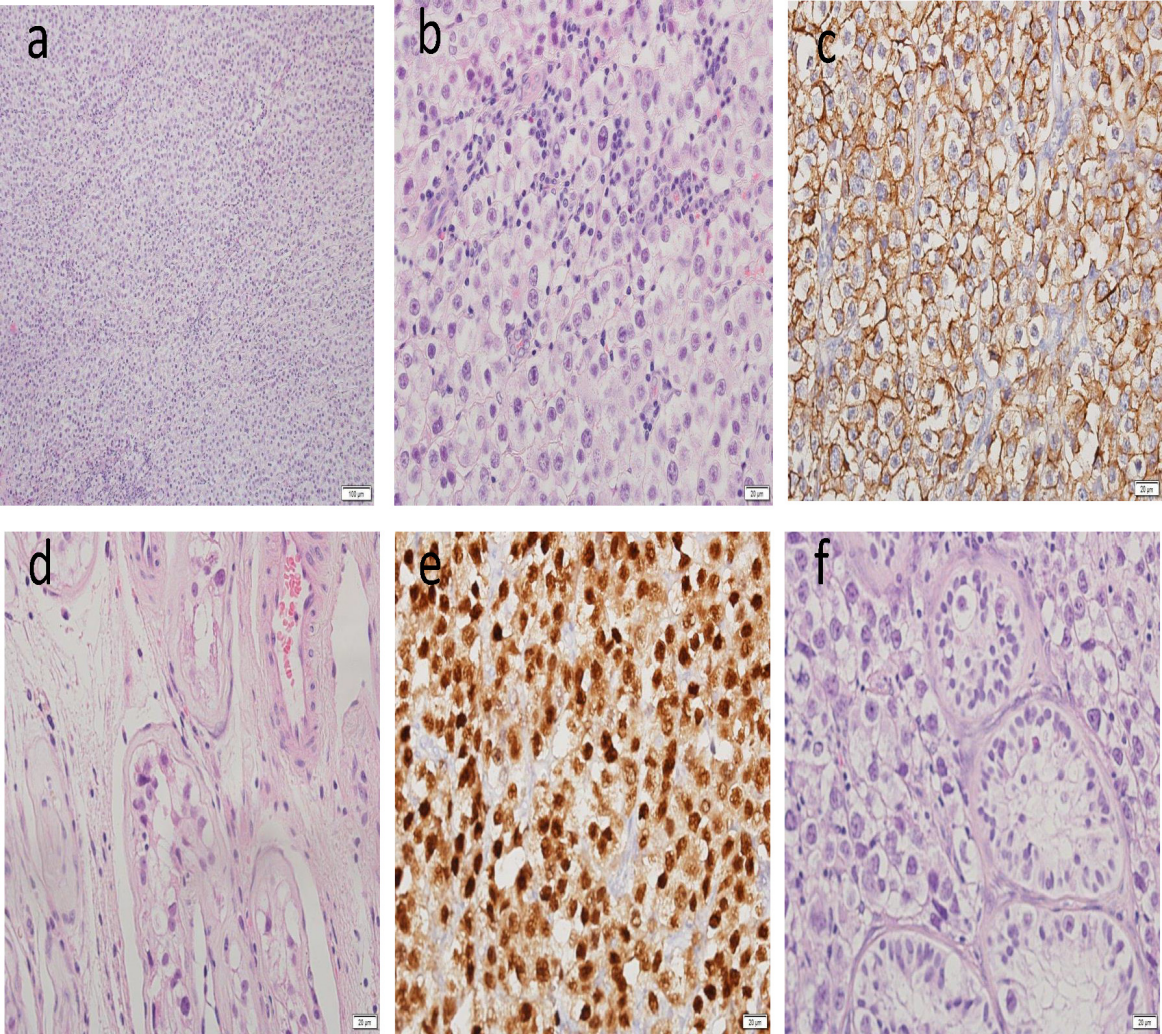
Cell morphology of seminoma by H&E staining and immuno‐stains. (a) General view × 10 (b) General view × 40 (c) c kit × 40 (d) ITGCN outside tumor × 40 (e) OKT4 × 40 (f) tumor with ITGCN at periphery × 40

First, we examined the cell morphology of the tumor by H&E and immuno‐stainings and confirmed its cell origin (Figure 1). In addition, we explored the possibility that normal cell line were the origin of the tumor, as a result of a germ line mosaicism. Two informative microsatellite polymorphic markers between the patient and his parents on chromosome 15 were analyzed in the patient's fibroblasts and iPSCs. The analysis revealed two maternal alleles but no paternal allele, confirming the earlier diagnosis of maternal UPD for chromosome 15 as the underlying mechanism for the PWS in this subject (Supporting Information Figure S1 and Figure S2). Identical allele compositions between the seminoma and the fibroblasts in both markers ruled out the possibility that normal cells have contributed to the development of a tumor in this patient as a consequence of mosaicism.

**Figure 2:**
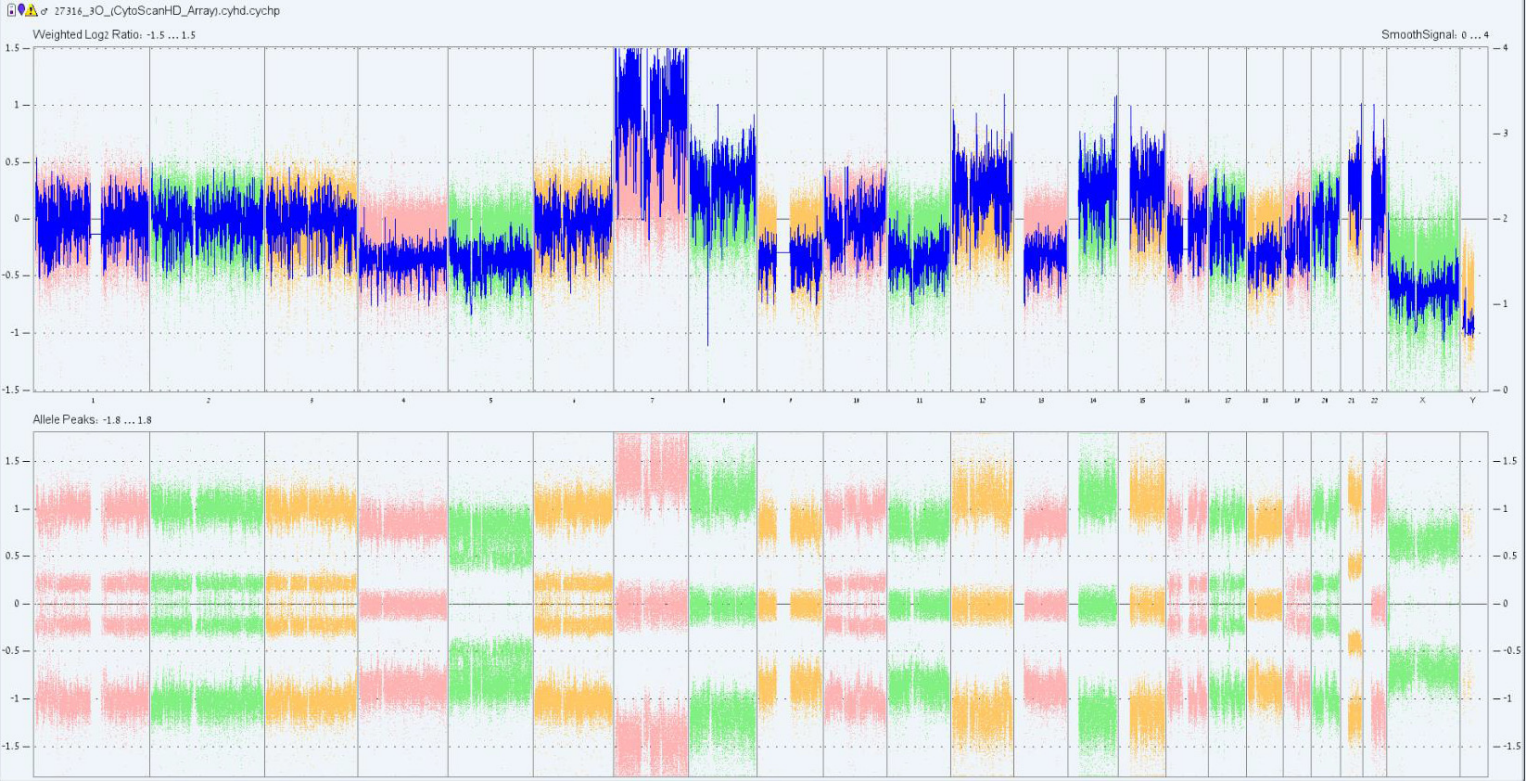
Chromosome analysis in seminoma by SNP array Chromosomes 1, 2, 3, 6, 10, 16, 17, 19, 20: mosaic monosomy of ~40%; Chromosomes 4, 9, 11, 13, 18: mosaic monosomy of ~50% with LOH of at least one population; Chromosome 5: mosaic monosomy with LOH of ~70%–80%; Chromosome 7: trisomy with LOH (unclear); Chromosomes 8, 12, 14, 15, 22: mosaic trisomy (unclear); Chromosome 21: trisomy in ~80% of cells; Chromosomes X, Y: normal

Next, we performed a CMA analysis from the tumor in order to determine chromosomal aberrations (Figure 2). We observed aneuploidy or mosaic aneuploidy in all chromosomes except chromosomes X and Y: for chromosomes no. 1, 2, 3, 6, 10, 16, 17, 19, 20 we found monosomy in a mosaic state of ~40% and for chromosomes no. 4, 9, 11, 13, 18–mosaic monosomy of ~50% with loss of heterozygosity (LOH) of at least one population. We also noted mosaic trisomy of 12p, 14, 15, and 22 and mosaic monosomy of at approximately 50% with LOH of at least one population of cells in chromosomes 7, 4, 9, 11, 13, and 18. Chromosome 5 exhibited a mosaic loss of approximately 75% with LOH in all cell populations.

**Figure 3:**
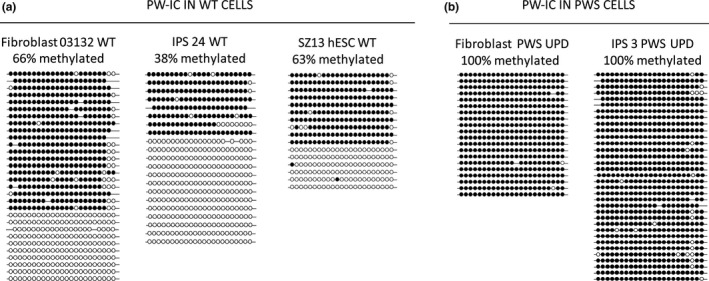
Methylation levels at the PWS‐IC in fibroblasts, iPS cells and seminoma cells of PWS patient by bisulfite DNA colony sequencing. Methylation levels at the PWS imprinting center (PW‐IC) as determined by bisulfite colony sequencing in: (a) wild type primary fibroblasts (Fibroblasts 03132 WT, 66%), iPSCs (IPS 24 WT, 38%) and human embryonic stem cell line (SZ13 hESC WT, 63%) controls, as compared to (b) patient's primary fibroblasts (Fibroblast PWS UPD, 100%) and iPSCs derived from them (IPS PWS UPD, 100%). Each line stands for a single DNA molecule. Full circles correspond to methylated CpGs while empty circles represent unmethylated CpGs

With this information in hand, we wished to explore whether the maternal imprints at the PWS locus (NC_000015.10) were maintained in the tumor considering the re‐establishment of parental imprints during the process of germ cell specification. For this we followed the methylation status of the PWS imprinting center (PW‐IC) region, which is normally methylated exclusively when transmitted by the mother, but not the father. Using bisulfite single colony sequencing, we verified the intermediate levels of DNA methylation at the PW‐IC that are expected in wild type fibroblasts and iPSCs derived from them. Methylation levels ranged from 38% to 66%, representing DNA molecules that are either completely methylated (maternally transmitted) or entirely unmethylated (paternally transmitted) (Figure 3a).

**Figure 4:**
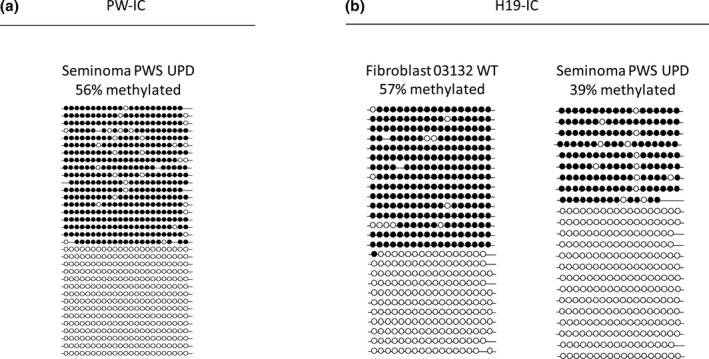
Methylation levels at the PW and H19 imprinting centers in seminoma. Methylation levels by bisulfite DNA colony sequencing at the: (a) PW‐imprinting center (PW‐IC) in patient's seminoma (Seminoma PWS UPD, 56%) and (b) H19‐imprinting centers (H19‐IC) in wild type fibroblasts (Fibroblast 03132 WT, 57%) and patient's seminoma (Seminoma PWS UPD, 39%). Each line stands for a single DNA molecule. Full circles correspond to methylated CpGs while empty circles represent unmethylated CpGs

Then, we determined the methylation status of this region in fibroblasts and induced pluripotent stem cells (iPSCs) derived from the patient (Figure 3b). Methylation levels were at their maximum (100%) in both cell types, as anticipated from maternal UPD for chromosome 15. However, they significantly decreased in the tumor, reaching 56% (Figure 4a). To understand the reasons underpinning the observed hypomethylation we extended our methylation study to the H19 imprinting center (H19‐IC), an additional locus that is under control of parental imprinting (methylated exclusively when transmitted by the father) (AF087017.1). By performing bisulfite colony sequencing for the H19‐IC region, we found intermediate methylation levels of 39% in the seminoma (Figure 4b), as would be expected if resetting of imprints in the germ line has not yet commenced.

## 4 DISCUSSION

In this paper, we described a pure, nearly diploid seminoma in an apparently infertile man with PWS due to maternal UPD on chromosome 15. Since males with PWS are infertile, the development of a germ cell tumor in this subject was an unanticipated result. We demonstrated that the maternal imprints at the PW‐IC were inappropriately maintained particularly in the tumor cells of this patient.

Primary testicular defect is the major contributor to hypogonadism in PWS males while hypogonadotrophic hypogonadism is less common (Hirsch et al., 2009). Sertoli cells secrete inhibin B and serum levels correlate with sperm counts in adult men. Inhibin B levels have been shown to be low to undetectable in all PWS men including those with hypogonadotrophic hypogonadism (Gross‐Tsur, Hirsch, Benarroch, & Eldar‐Geva, 2012) (Hirsch et al., 2009). Although testicular biopsies in PWS infants and prepubertal boys showed variable histologic findings including presence of normal Sertoli cells and germ cells (Vogels, Moerman, Frijns, & Bogaert, 2008), (Hirsch, Eldar‐Geva, Erlichman, Pollak, & Gross‐Tsur, 2014) (Bakker, Wolffenbuttel, Looijenga, & Hokken‐Koelega, 2015), fertility has never been published in PWS males, contrary with females, suggesting that spermatogenesis is markedly impaired in adult PWS men.

To explain the apparent contradiction between the existence of a germ cell tumor and hypogonadism in this patient, we first confirmed germ cell origin of the tumor by histopathology and found total absence of any normal testicular tissue. Next, we determined the tumor chromosomal composition and found extensive chromosomal instability, exhibiting aberrations that are typical to seminomas (such as acquisition of trisomy 12p, gain of chromosomes 7, 8 and loss of 11, 13, and 18) (Looijenga et al., 2000) (Ottesen et al., 1997).

By showing identical haplotype analysis on DNA from the tumor the patient's skin fibroblasts and iPSCs derived from them, we confirmed the presence of the maternal UPD in all examined cell types and ruled out the possibility that normal cells might have been the origin of the tumor as a consequence of germ line mosaicism.

Finally, we looked to see if the maternal imprints at the PWS locus were maintained in the tumor since re‐establishment of parental imprints may occur during the process of germ cell specification**.** Methylation levels were reduced to 50% in the tumor cells, while the expected full methylation pattern was preserved in fibroblasts and iPSCs. The intermediate methylation levels that were observed in the tumor may represent incomplete resetting of maternal imprints while undergoing cell specifications in the male genital tract. On the other hand, it may simply result from relaxation of parental imprints as a consequence of transformation, a feature that is typical to germ cell tumors.

We speculated that the tumor may have developed from a primordial germ cell at the time when the epigenetic marks were erased before they were re‐established in the male germ line. Indeed, methylation analysis of the imprinting control region of PWS apparently supports this possibility since the maternal imprints, which are stringently preserved in the soma and even during cell reprogramming into iPSCs (100% methylation), drop to nearly 50% in the tumor, correlating with the erasure of parental imprints during PGC migration and development. On the other hand, relaxation of imprinted methylation, which has been found in the majority of GCTs (Schneider et al., 2001), might be related to the process of malignant transformation rather than reflecting the cell origin of the tumor.

To discriminate between both possibilities we extended our analysis to an unrelated locus that is also under the regulation of genomic imprinting, the H19‐IC. The H19‐IC is located on 11p15 and is regulated by differential methylation but in a reciprocal fashion; it is methylated when paternally transmitted while unmethylated when maternally transmitted. As such, it is predicted to be completely methylated following the removal of the mother's imprints, or entirely unmethylated following the re‐establishment of the father's imprints. H19‐IC, as opposed to the PW‐IC, is hypomethylated in virtually all seminomas with the exception of spermatocytic seminomas which originate from mature germ cells, rather than from a primordial germ cell (Sievers et al., 2005). Hence, we used its methylation status as a marker for the erasure of parental imprints during PGC specification. The methylation of H19‐IC remained unchanged (~50%) designating that reprogramming of the imprints have not yet begun in the cell origin of the tumor. Therefore, we infer from this assay that the loss of methylation in the PW‐IC specifically in the tumor of our patient is most likely a locus‐specific event resulting from imprint relaxation rather than representing a more general phenomenon of resetting parental imprint genome wide which normally occurs during germ line specification.

It should be noted that the finding of this research cannot permit to determine the order of events or precise timing of imprint relaxation during tumorigenesis. This is since we cannot completely rule out the possibility of a small, nonetheless significant, contribution of cells from the ITGCN component (premelignant stage) to the tumor. In addition, taking into consideration that the tumor is comprised of other cell types besides pure seminoma, it is anticipated that the extent of demethylation is even greater in practice. This is since all the cells in the body of our PWS patient are completely methylated (100%) at the PWS‐IC due to maternal UPD for chromosome 15 (see primary fibroblasts from patient, Figure 3). Hence, it is anticipated that the contribution of such nontumorigeneic cells (completely methylated at the disease related locus) would have an opposite outcome by diluting the effect of imprint relaxation and therefore demethylation at the disease‐related locus.

Altogether, by carrying out comprehensive analysis on the methylation status of his GSC, we infer that the development of a germ cell tumor in PWS cannot be attributed to the resetting of the methylation imprints at the PW‐IC, as originally proposed.

In conclusion: Unexpectedly we found pure seminoma in a man with PWS who suffers from hypogonadotrophic hypogonadism. Methylation levels were reduced to nearly 50% in the tumor, in contrast with complete methylation of his fibroblasts and pluripotent stem cells. We documented that the loss of methylation in the PW‐IC in the tumor is a locus‐specific event resulting from imprint relaxation rather than, a more general phenomenon, of resetting parental imprint genome wide which normally occurs during germ line specification.

## AUTHOR CONTRIBUTIONS

G.A., R.S., S.Z., E.G., and S.E.L. contributed to the conception and design of the study, the collection and assembly of the data, data analysis and interpretation. T.E.G., V.G.T., and H.J.H. contributed to the data analysis and manuscript writing. R.E. contributed to the conception and design of the study, financial support, data analysis and interpretation, and manuscript writing.
