# Supplementary figures and images for: Incomplete methylation of a germ cell tumor (Seminoma) in a Prader‐Willi male
Source: Mol Genet Genomic Med. 2018 Jul 12;6(5):811–8. doi: 10.1002/mgg3.448 (PMC6160713; doi:10.1002/mgg3.448)

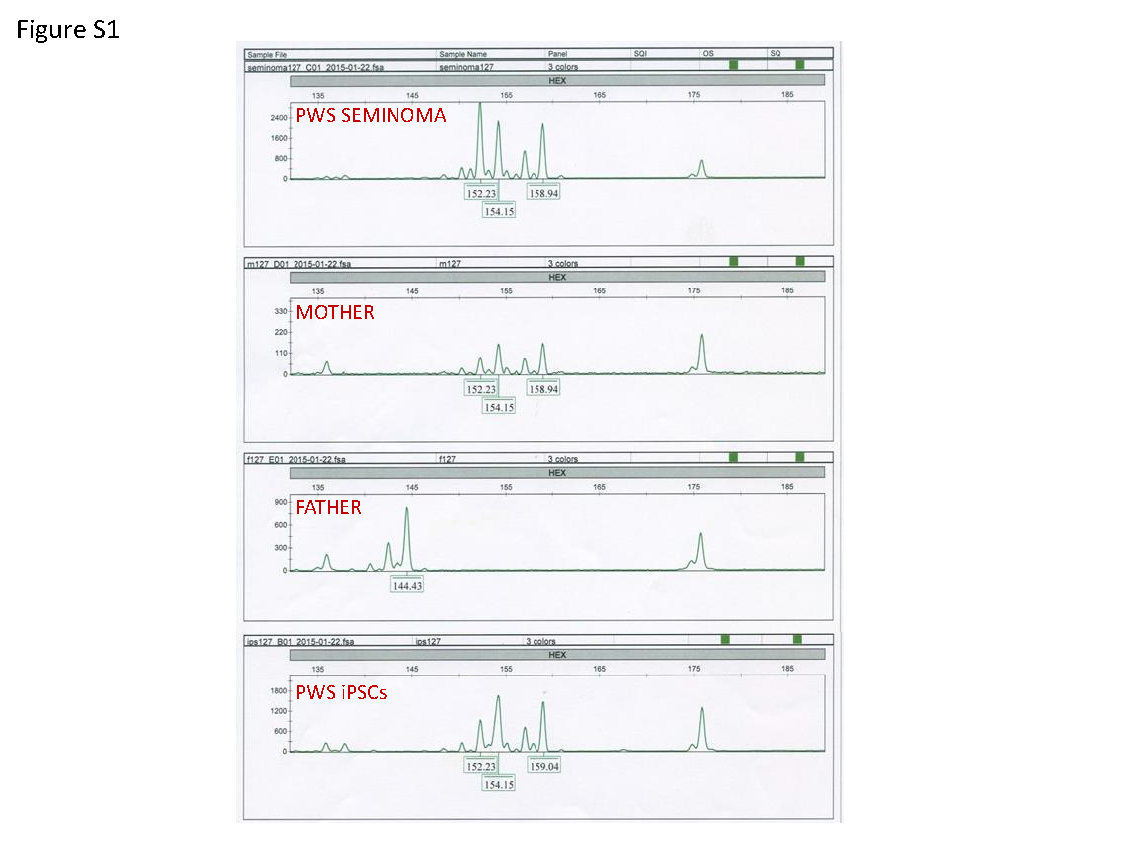

Supplement: Supplementary file 1 [file MGG3-6-811-s001.tif]

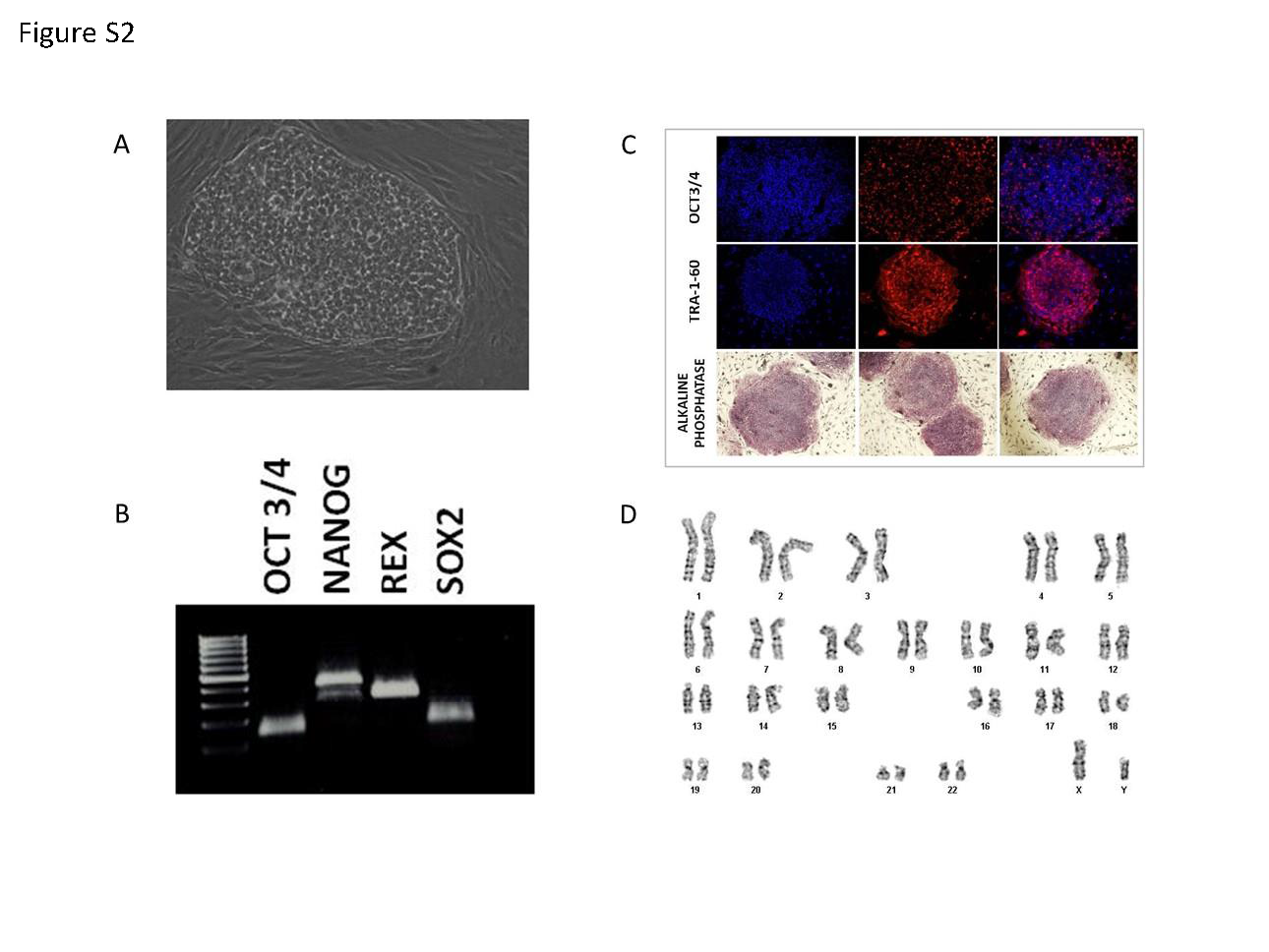

Supplement: Supplementary file 2 [file MGG3-6-811-s002.tif]
